# Identification and characterization of N6-methyladenosine circular RNAs in the spinal cord of morphine-tolerant rats

**DOI:** 10.3389/fnins.2022.967768

**Published:** 2022-08-05

**Authors:** Manyu Xing, Meiling Deng, Yufei Shi, Jiajia Dai, Tong Ding, Zongbin Song, Wangyuan Zou

**Affiliations:** ^1^Department of Anesthesiology, Xiangya Hospital, Central South University, Changsha, China; ^2^National Clinical Research Center for Geriatric Disorders, Xiangya Hospital, Central South University, Changsha, China

**Keywords:** morphine tolerance, circRNAs, N6-methyladenosine, microarray, bioinformatics analysis

## Abstract

Morphine tolerance (MT) is a tricky problem, the mechanism of it is currently unknown. Circular RNAs (circRNAs) serve significant functions in the biological processes (BPs) of the central nervous system. N6-methyladenosine (m^6^A), as a key post-transcriptional modification of RNA, can regulate the metabolism and functions of circRNAs. Here we explore the patterns of m^6^A-methylation of circRNAs in the spinal cord of morphine-tolerant rats. In brief, we constructed a morphine-tolerant rat model, performed m^6^A epitranscriptomic microarray using RNA samples collected from the spinal cords of morphine-tolerant rats and normal saline rats, and implemented the bioinformatics analysis. In the spinal cord of morphine-tolerant rats, 120 circRNAs with different m^6^A modifications were identified, 54 of which were hypermethylated and 66 of which were hypomethylated. Functional analysis of these m^6^A circRNAs found some important pathways involved in the pathogenesis of MT, such as the calcium signaling pathway. In the m^6^A circRNA-miRNA networks, several critical miRNAs that participated in the occurrence and development of MT were discovered to bind to these m^6^A circRNAs, such as miR-873a-5p, miR-103-1-5p, miR-107-5p. M^6^A modification of circRNAs may be involved in the pathogenesis of MT. These findings may lead to new insights into the epigenetic etiology and pathology of MT.

## Introduction

Morphine, a traditional opioid analgesic, plays an important role in managing acute and chronic pain (Wiffen et al., 2016). However, chronic morphine administration often leads to the reduction of analgesic efficacy which is known as morphine tolerance (MT). MT is still a difficult issue; and it often leads to the withdrawal of treatment and many side effects, including physical dependence, constipation, vomiting, respiratory depression, and sometimes even death due to dose escalation (Paul et al., 2021). Multiple dysfunctions of biological processes (BPs) involved in the development of MT have been proposed (Chen and Sommer, 2009; Uniyal et al., 2020; Gledhill and Babey, 2021), including the opioid receptor desensitization, downregulation, internalization (Williams et al., 2013), and altered epigenetic regulation (Liang et al., 2013; Viet et al., 2017). Despite significant headway in this field, the mechanisms underlying the development of MT were not well understood.

The recent focus on the mechanism of many diseases is the role of non-coding RNAs (ncRNAs). Our previous studies reported the shreds of evidence of ncRNAs, including microRNAs (miRNAs) (Huang et al., 2019b), long non-coding RNAs (lncRNAs) (Shao et al., 2018), and circRNAs (Weng et al., 2019) in the regulation of gene expression in the spinal cord of morphine-tolerant rats. The role of miR-873a-5p (Huang et al., 2019a), and lncRNA MRAK159688 (Deng et al., 2022) have already been confirmed. CircRNAs are widely expressed in the central nervous system (Rybak-Wolf et al., 2015), they are stable and highly conserved (Jeck et al., 2013). They can regulate transcription and translation by sponge miRNAs or interacting with proteins. With these characteristics, circRNAs hold significant functions as diagnostic biomarkers and therapeutic targets for diseases, including MT (Lu et al., 2019; Mehta et al., 2020). We had previously built circRNA expression profiles in the spinal cord of morphine-tolerant rats (Weng et al., 2019). However, little is known about the upstream regulatory mechanism of circRNAs modification in MT.

M^6^A is one of the most common mRNA epitranscriptomic modifications (Niu et al., 2013; Meng et al., 2019), it refers to the N6 position of the adenine base is modified by a methyl group. In addition to mRNA, m^6^A modification also occurs in circRNAs (Ma et al., 2019; Wang et al., 2021; Wu et al., 2021). There are three factors regulating the m^6^A methylation: “writers,” “erasers,” and “readers.” The roles of these factors are as the name suggests (Zaccara et al., 2019). “Writers” are methyltransferases, including methyltransferase-like 3 and 14 proteins (METTL3 and METTL14) and their cofactors WT1 associated protein (WTAP), etc. “Readers” are RNA binding proteins that can specifically read m^6^A methylation, YTH domain-containing proteins (YTHDC1, YTHDC2, YTHDF, etc.) were identified as potential m^6^A-binding proteins; “Erasers” are demethylases, including fat mass and obesity-associated protein (FTO) and AlkB homologue 5 (ALKBH5). The m^6^A modification can affect many aspects of post-transcriptional processes of RNA, including splicing, translational efficiency, nuclear exporting, and RNA structure (Ma et al., 2019). The m^6^A modification in circRNAs displayed cell-type-specific methylation patterns, making m^6^A circRNAs more valuable in diagnosing and treating diseases (Zhou et al., 2017).

The epigenetic role of m^6^A circRNA in the pathogenesis of MT has not been reported. Here we hypothesized that m^6^A circRNAs might be associated with MT. We built an epitranscriptomic map using microarray analysis and explored the potential functions of m^6^A circRNAs in the spinal cord of morphine-tolerant rats, and the results might provide novel clues for epigenetic etiology and pathogenesis of MT.

## Materials and methods

### Animal model and behavior tests

Adult Sprague-Dawley (SD) male rats weighing 240–260 g were obtained from the Hunan SJA Laboratory Animal Company (Hunan, China). The animals were housed in specific pathogen-free and individually ventilated cages in a room with a 12 / 12 light-dark cycle and a temperature of 22 ± 1°C. We used the same procedure of our previous research to establish a rat model of MT (Weng et al., 2019). Briefly, the adult male SD rats were randomly divided into the normal saline group (NS group) and MT group. Rats in the MT group were administrated morphine hydrochloride injection of 10 μg / 10 μl intrathecally through the tube inserted into the sheath at 8:00 a.m. and 8:00 p.m. for 7 consecutive days, the NS group was given 10 μl of normal saline at the same time points. The tail-flick test was carried out under the condition that the water temperature allowed the basal latency of 4–6 s, and the cutoff time was set to 10 s to minimize tissue damage. The tail-flick time was converted into the percentage of maximal possible antinociceptive effect (%MPE). All animal experimental procedures were approved by the Ethics Committee for Animal Experimentation of Central South University and followed the Chinese National Institutes of Health Guide for the Care and Use of Laboratory Animals.

### RNA sample isolation and quality control

On the morning of the 8th day, 1 h after morphine or normal saline injection, rats were fully anesthetized with 1% pentobarbital (50mg/kg, i.p.) and sacrificed. The lumbar enlargements of spinal cords were surgically collected and were refrigerated at –80°C until used. The total RNA of lumbar enlargements was extracted following the standard protocol of TRI Reagent (Sigma, T9424), and the RNA quantity and quality were tested through NanoDrop ND-1000 Spectrophotometer (Thermo Fisher Scientific, the United States). RNA integrity and gDNA contamination were tested by denaturing agarose gel electrophoresis.

### Quantitative (real-time) polymerase chain reaction

The mRNA expression of m^6^A-related enzymes, including METTL3, METTL14, WTAP, FTO, ALKBH5, YTHDC2, YTHDF1, and YTHDF2 was detected separately by qRT-PCR to investigate the m^6^A methylation status in the spinal cord of morphine-tolerant rats. In brief, the extracted total RNAs were reverse transcribed to cDNA using Prime Script RT Reagent Kit (Takara, China) according to the manufacturer’s instructions. The primer sequences for m^6^A-related enzymes are shown in Supplementary Table 1. Real-time PCR reaction using PCR master mix (Arraystar, AS-MR-006-5) was performed according to the manufacturer’s manual in the ViiA7 Real-Time PCR Detection System (Applied Biosystems, the United States).

### N6-methyladenosine immunoprecipitation and N6-methyladenosine circular RNAs microarrays

The sample preparation and microarray hybridization were performed based on Arraystar’s standard protocols. Briefly, the total RNAs were immunoprecipitated with the anti-m^6^A antibody (Synaptic Systems, 202003). The modified RNAs were eluted from the immunoprecipitated magnetic beads as the “IP.” The RNAs contained in the supernatant were unmodified as the “SUP.” The “IP” and “SUP” RNAs were treated with RNase R (Epicenter, RNR07250) separately to enrich circRNAs, and then labeled with Cy5 and Cy3, respectively, as cRNAs using Arraystar Super RNA Labeling Kit (Arraystar, AL-SE-005). The labeled cRNAs were combined and hybridized to Arraystar Rat CircRNA Epitranscriptomic Arrays (8x15K, Arraystar) at 65°C for 17 h in an Agilent Hybridization Oven. After washing the arrays were scanned in two-color channels by an Agilent Scanner G2505C (Agilent, the United States). The raw RNA intensities of IP (Cy5-labeled) and SUP (Cy3-labeled) were analyzed by Agilent Feature Extraction software (version 11.0.1.1) and normalized with an average of log2-scaled spike-in RNA intensities.

The normalized IP intensities represent the “m6A quantity” of the transcript comparable across samples. The m^6^A methylation levels were calculated as the percentage of modification (IP normalized intensities) of the input (IP and SUP normalized intensities). We also calculated the expression levels of circRNAs based on the normalized IP and SUP intensities. We used the criteria (fold change ≥ 1.5 and *P* < 0.05) to screen the differentially m^6^A-methylated or expressed circRNAs between the two groups.

### Methylated RNA immunoprecipitation and quantitative real-time polymerase chain reaction

To validate the microarray data, the IP and SUP RNAs from the eight spinal cords were separately reverse transcribed and performed qRT-PCR. The primers of the circRNAs are shown in Supplementary Table 1. The fraction of IP in the input was calculated using the following formula as %MERIP/Input = $\frac{2^{-\text{CtIP}}}{2^{-\text{CtIP}}+2^{-\text{CtSUP}}}\times 100\%$.

### Bioinformatics analysis

Hierarchical clustering was performed by Cluster 3.0 to show the distinguishable m^6^A-methylation pattern among samples, and the heat maps were generated in Java Treeview. The host genes of the differentially deregulated m^6^A-circRNAs were analyzed according to the pathway annotations of the Kyoto Encyclopedia of Genes and Genomes (KEGG) and Gene Ontology (GO) functional enrichment using the DAVID 6.8.^1^ The correlation and the cumulative analysis between methylation and the expression level were performed in R v4.1.0 software. The upset graph showing the distribution patterns of circRNAs in terms of m^6^A quantity, m^6^A methylation levels, and expression was created using online tools.^2^ Potentially targeted miRNAs by circRNAs were predicted with Arraystar’s home-made miRNA target prediction software based on TargetScan and miRanda. The predicted circRNA/miRNA interaction networks were generated to visualize the interactions by Cytoscape (version 3.8.0).

### Statistical analysis

For behavior data, two-way ANOVA followed by Bonferroni’s post-hoc test was applied to assess the differences over time. For qRT-PCR statistical analyses, a two-tailed unpaired Student’s *t*-test was used for two-group comparisons in GraphPad Prism 9.0 software (La Jolla, CA). *P* < 0.05 was considered statistically significant.

## Results

### Morphine-tolerant rat model

**FIGURE 1:**
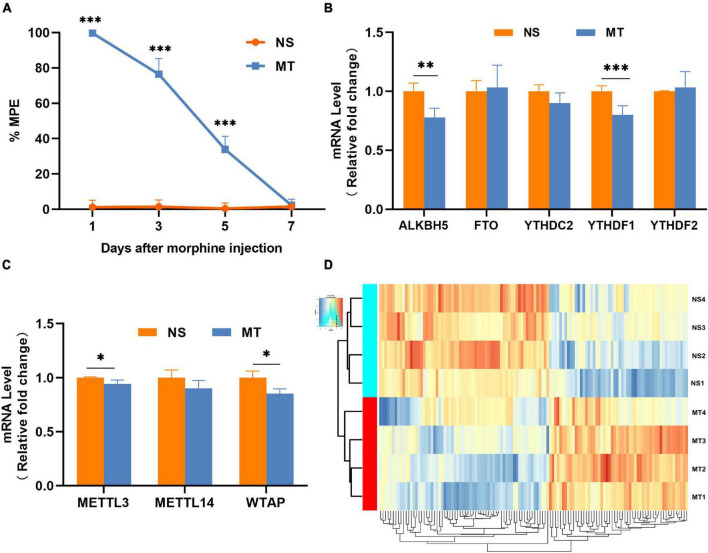
The animal model and the overall features of m^6^A-circRNA in morphine-tolerant rats. **(A)** Morphine-induced antinociception was assessed through the tail-flick test, and tail-flick latency was converted to %MPE (*n* = 4), ***P < 0.001 compared with the NS group using Two-way repeated measures of ANOVA followed by Bonferroni’s post-hoc test. **(B)** Expression of RNA demethylase and methyltransferase determined by qRT-PCR. (*n* = 4) **P < 0.01, ***P < 0.001 compared with the NS group using a two-tailed unpaired Student’s *t*-test. **(C)** Expression of RNA methylase determined by qRT-PCR (*n* = 4). *P < 0.05 compared with the NS group using a two-tailed unpaired Student’s *t*-test. **(D)** Hierarchical clustering shows global m^6^A-methylated circRNAs between the MT group and NS group (*n* = 4).

After repeated intrathecal administration of morphine for seven consecutive days, the %MPE dropped from 100% to almost 0%, and the morphine-tolerant rat model was constructed successfully (Figure 1A). In the spinal cord of morphine-tolerant rats, the mRNA expressions of m^6^A “eraser” (FTO and ALKBH5), m^6^A “readers” (YTHDC2, YTHDF1, and YTHDF2) (Figure 1B), and m^6^A “writers” (METTL3, METTL14, and WTAP) (Figure 1C) were tested separately, we found that the expressions of METTL3, WTAP, ALKBH5, and YTHDF1 were downregulated in the MT group compared with the NS group. These dysregulations of m^6^A “writers,” m^6^A “eraser,” and m^6^A “readers” may lead to a dynamic change of m^6^A methylation in the spinal cord of morphine-tolerant rats.

### Overview of the differently methylated circular RNAs

**FIGURE 2:**
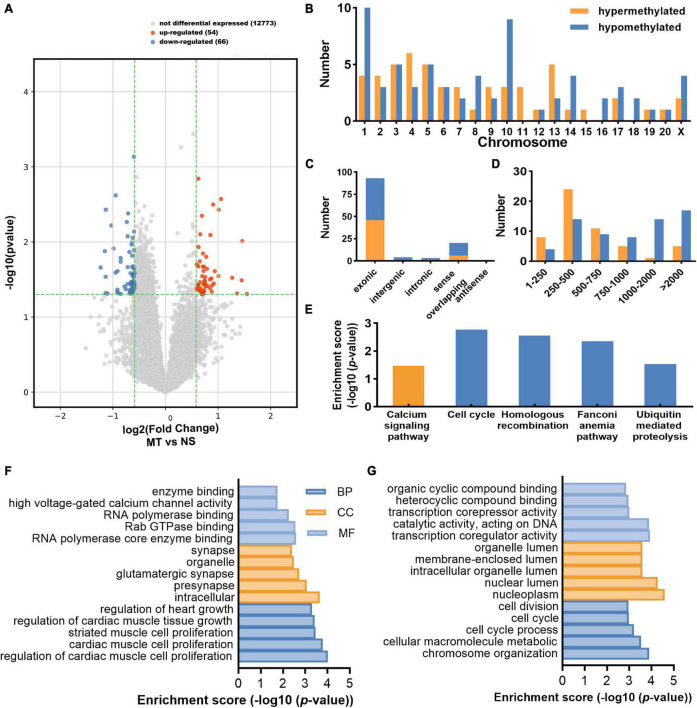
Distribution of differentially methylated circRNAs. **(A)** The volcano plot shows the distribution of differentially methylated circRNAs (Fold change ≥ 1.5 and *P* < 0.05) between the MT group and the NS group. **(B)** The chromosome’s origins for host genes of differentially m^6^A-methylated circRNAs. **(C)** The genomic origins of differentially m^6^A-methylated circRNAs. **(D)** The length distribution of differentially m^6^A-methylated circRNAs. **(E)** The enriched pathways of the host gene of the differentially m^6^A-methylated circRNAs through KEGG analyses. **(F)** The top 15 enriched GO terms of the host gene of the hypermethylated circRNAs. GO terms include biological process (BP) analysis, cellular component (CC) analysis, and molecular function (MF) analysis. **(G)** The top 15 enriched GO terms of the host gene of the hypomethylated circRNAs.

The m^6^A-circRNA microarrays were performed in the spinal cords of eight biological replicates from the morphine-tolerant and control rats (Figure 1D). All microarray results were uploaded to the GEO database (GSE199892). A total of 12,669 m^6^A-circRNAs were identified, compared with the control group, 120 circRNAs with different m^6^A modifications were identified, 54 m^6^A-circRNA were hypermethylated and 66 m^6^A-circRNAs were hypomethylated in the MT group (Figure 2A). The top 10 significantly methylated circRNAs are shown in Table 1.

**TABLE 1:** The detailed information of significantly top 10 hypermethylated m^6^A-circRNA and top 10 hypomethylated m^6^A-circRNAs.

circRNA	Regulation	Fold change	*P*-value	Gene symbol
rno_circRNA_005515	Hyper	2.92708	0.04929	Zdhhc20
rno_circRNA_011473	Hyper	2.75185	0.00975	Suclg2
rno_circRNA_002997	Hyper	2.73076	0.03240	Ttc3
rno_circRNA_006947	Hyper	2.56731	0.04853	LOC100360412
rno_circRNA_012089	Hyper	2.41173	0.02999	Mkln1
rno_circRNA_012088	Hyper	2.08241	0.00268	Mkln1
rno_circRNA_008556	Hyper	2.01664	0.00373	Mnd1
rno_circRNA_010013	Hyper	2.00984	0.02843	Rtf1
rno_circRNA_011501	Hyper	1.91584	0.02445	Shq1
mmu_circRNA_017077	Hyper	1.88999	0.03587	Mga
rno_circRNA_012261	Hypo	0.42555	0.02187	Fam126a
rno_circRNA_014500	Hypo	0.45007	0.01327	None
mmu_circRNA_45606	Hypo	0.45484	0.04811	Pdk3
rno_circRNA_004940	Hypo	0.45499	0.02904	Pds5a
mmu_circRNA_34955	Hypo	0.45590	0.00371	Zmynd8
rno_circRNA_002718	Hypo	0.46618	0.04869	Nmt1
rno_circRNA_015839	Hypo	0.49006	0.00602	Map4
rno_circRNA_011759	Hypo	0.50429	0.01225	Aebp2
rno_circRNA_000444	Hypo	0.51787	0.02561	RGD1310712
rno_circRNA_018043	Hypo	0.51866	0.00239	Brwd3

The different methylated (DE) m^6^A circRNAs were transcribed from all chromosomes, the host genes of hypermethylated circRNAs were primarily located in chromosomes 4 (6), 5 (5), and 13 (5), while the hypomethylated parts were mostly located in chromosome 1 (10), 3 (5), 5 (5), and 10 (9) (Figure 2B). Furthermore, most of the DE m^6^A-circRNAs were encoded by the exonic sequence of the host genes (Figure 2C). Their lengths were mostly enriched in 1–10,000 bps (Figure 2D).

To explore the physiological and pathological functions of m^6^A-circRNAs in MT, we performed GO and KEGG analysis for the host genes of DE m^6^A –circRNAs. The host genes of hypermethylated circRNAs were most enriched in the calcium signaling pathway. The host genes of hypomethylated circRNAs were most enriched in the cell cycle pathway, homologous recombination pathway, fanconi anemia pathway, ubiquitin mediated proteolysis pathway (Figure 2E). The GO terms of hypermethylated circRNAs were presented in Figure 2F. The GO terms were most enriched in regulation of cardiac muscle cell proliferation in the BP term; intracellular in the cellular component (CC) term; RNA polymerase core enzyme binding in molecular functions (MF) term. For the hypomethylated circRNAs, the GO terms were most enriched in chromosome organization in BP term, nucleoplasm in CC term, and transcription coregulator activity in MF term (Figure 2G).

Besides, the m^6^A modification levels of these 12,699 circRNAs were calculated as the percentage of modified RNA in all input RNAs (Supplementary Figure 1A). In total, we identified that 464 circRNAs had significantly differential modification levels in the MT group. Surprisingly, among these 464 circRNAs, only 1 circRNA had upregulated methylation level, and 463 circRNAs had downregulated methylation level (Supplementary Figure 1B).

### Combined analysis of N6-methyladenosine methylation and expression of circular RNAs

**FIGURE 3:**
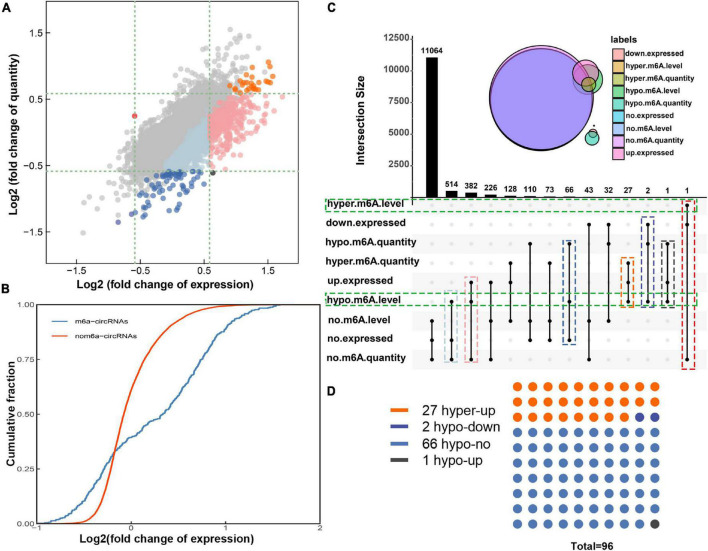
Joint analysis of m^6^A methylation and expression patterns of circRNAs in morphine tolerance. **(A)** The nine-quadrant graph shows the relationship between m^6^A quantity and the expression of circRNAs. **(B)** Cumulative distribution of circRNAs expression between MT group and NS group for m^6^A-circRNAs (blue) and non-m^6^A- circRNAs (red). **(C)** The upset diagram shows the m^6^A quantity, expression, and m^6^A level of circRNAs and presents 14 different methylation and expression patterns of circRNAs. **(D)** The dot plot shows the distribution of 96 circRNA with both dysregulated m^6^A quantity and m^6^A level. The orange dots (hyper-up) show 27 circRNAs were hypermethylated and up-expressed, 2 navy blue dots (hypo-down) represent circRNAs were hypomethylated and down-expressed; 1 black dot (hypo-up) represent circRNA were hypomethylated and up-expressed; the light blue dots (hypo-no) represent 66 circRNAs with decreased m6A modification quantity but unchanged expression levels.

By conjoint analysis of the results of m^6^A methylation and expression of circRNAs in morphine-tolerant rats, we found that 155 hypermethylated circRNAs were upregulated, 1 hypermethylated circRNA was downregulated, and 34 hypomethylated circRNAs were downregulated (Figure 3A). To further analyze how m^6^A methylation affects circRNA expression, 12,669 m^6^A-circRNAs were divided into two groups, 439 in the m^6^A-circRNAs group and 12,230 circRNAs in the non-m^6^A-circRNAs group. Then we calculated the log_2_ fold change of expression of them and generated cumulative curves. We found that the proportion of circRNAs not modified by m^6^A was higher than that of circRNAs modified by m^6^A in terms of the log2FC of the circRNA between 0 and 1.5 (Figure 3B).

However, studies have shown that the potential effects of RNA modifications depend not only on the m^6^A quantity but also on the percentage of transcripts that are modified. Therefore, the combined analysis of m^6^A level and expression of circRNAs was also been performed (Supplementary Figure 1C). Moreover, we studied the m^6^A quantity and m^6^A methylation level of all the 12,966 circRNAs. The upset graph showed all the circRNAs distribution in 14 different patterns considering m^6^A quantity, expression level and m^6^A methylation level (Figure 3C). Only one circRNA had increased m^6^A modification level with unchanged m^6^A quantity and decreased expression level. In addition, a total of 896 circRNAs with hypomethylated m^6^A level but with unchanged m^6^A quantity were also identified. In both cases, we need to be wary of false-positive in m^6^A levels simply due to altered expression levels (Figure 3C).

We focused on the remaining 96 circRNAs with hypomethylated m^6^A level. In detail, 27 circRNAs were hypermethylated and up-expressed, and 2 circRNA was hypomethylated and down-expressed; this may suggest that the m^6^A modification on these circRNAs may promote RNA stability. 1 circRNAs were hypermethylated and down-expressed; this prompted the m^6^A modification may inhibit circRNA stability and promote circRNA degradation. Furthermore, there were 66 circRNAs with decreased m^6^A modification quantity but unchanged expression levels; this suggests that m^6^A modifications in these circRNAs do not affect their expression and may play other roles, such as post-translational regulation (Figures 3C,D).

### Construction of N6-methyladenosine circular RNAs-microRNAs networks in morphine tolerance

**FIGURE 4:**
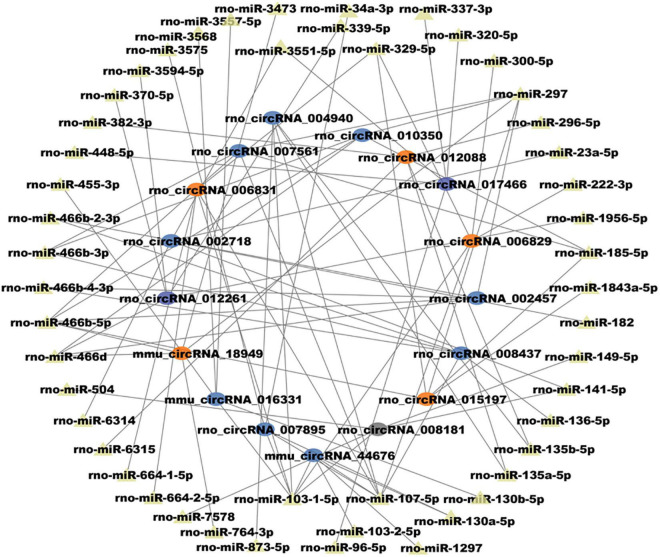
Construction of circRNA–miRNA networks in morphine tolerance using circRNAs with differences in both m^6^A quantity and m^6^A modification level.

**FIGURE 5:**
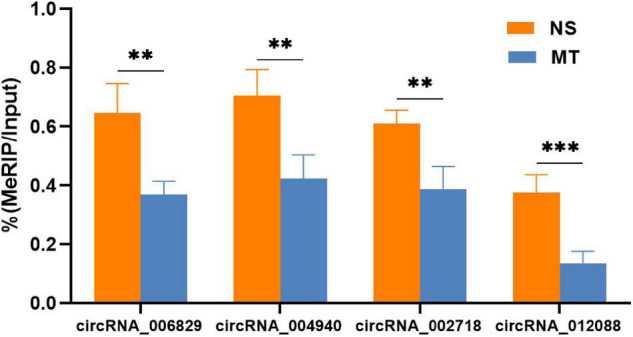
M^6^A levels of the four circRNAs involved in the circRNA–miRNA networks were verified by MeRIP-qPCR. Relative m^6^A methylation level was calculated as the percentage of modified transcripts in all transcripts. *P*-values were calculated using Student’s *t*-test (*n* = 4). **P < 0.01, ***P < 0.001 compared with the NS group using a two-tailed unpaired Student’s *t*-test.

Given the importance of circRNA–miRNA interaction and to further explore the underlying mechanism of the above m^6^A-circRNAs, circRNAs with differences in m^6^A quantity and m^6^A level were selected to construct circRNA–miRNA networks. Several significant miRNAs that participated in the occurrence and development of MT were found to bind to these m^6^A circRNAs (Figure 4), such as miR-873a-5p, miR-103-1-5p, and miR-107-5p (Dai et al., 2018; Huang et al., 2019b). These data suggest that circRNAs with DEm^6^A might play a role in the pathological process of MT. Finally, to further confirm the findings of the m^6^A microarray data, we analyzed the m^6^A modification level of four circRNAs using MERIP-qPCR. The results indicate that the methylation levels (%MeRIP / Input) of these cirRNAs were downregulated and were consistent with the microarray analysis (Figure 5).

## Discussion

In this study, we identified the epitranscriptomic map of m^6^A circRNAs in the spinal cord of morphine-tolerant rats. Specifically, we identified a total of 120 circRNAs with different m^6^A modifications including 54 hypermethylated circRNAs and 66 hypomethylated circRNAs, and found the host genes of these m^6^A circRNAs were involved in the key process of MT, such as the calcium signaling pathway. Meanwhile, we researched the correlation among m^6^A-methylation quantity, expression, and m^6^A level of circRNAs, and found 14 modifications and expression patterns. This study provides a deeper understanding of the functional role of circRNAs in MT and brought novel clues for epigenetic etiology and pathogenesis of MT.

The m^6^A modification status is probably associated with the expression of m^6^A “writers,” “readers,” and “erasers.” Research showed that METTL3 could regulate the global m^6^A level in the spinal cord of inflammatory pain (Pan et al., 2021). In our study, compared with the NS group, the expression levels of METTL3, WTAP, ALKBH5, and YTHDF1 decreased significantly in the MT group. Due to the imbalance between methyltransferase and demethylase in MT, m^6^A methylation microarrays resulted in both hypermethylated and hypomethylated circRNAs in the spinal cord of MT. Further research is needed to verify their specific roles and functions targeting a particular circRNA.

The chronic morphine administration could cause adaptive changes in neurons and neuronal communications (Sanna et al., 2020; Uniyal et al., 2020). The molecular adaptations include the dynamic change of opioid receptors, ion channels etc. (Williams et al., 2013). In the present study, the function analysis of DE m^6^A circRNAs enriched in the calcium signaling pathway. CACNA1B (Cav2.2) is one of the key genes in this pathway, it encodes an N-type voltage-gated calcium channel ubiquitously that is important in regulating neuropathic pain (Gao et al., 2021). Previous studies have demonstrated that increased intracellular levels of Ca^2+^ in neurons was an essential component of MT (Smith et al., 1999). Ca^2+^ channel antagonists enhanced morphine analgesia and prevented the development of tolerance (Michaluk et al., 1998). In addition, our previous study found that CaMKIIγ expression in the spinal cord was gradually upregulated after chronic morphine treatment, and downregulated it could alleviate tolerance (Wang et al., 2017). This evidence supports the hypothesis that m^6^A modification of circRNAs participated in the progression of MT.

Research has shown that m^6^A modification could regulate mRNA transcription, splicing, and degradation thus influencing its expression (Meyer and Jaffrey, 2014; Yue et al., 2015). Zhou et al. (2017) found low m^6^A level was negatively associated with circRNAs expression, while high m^6^A level was not linked to circRNAs expression in human embryonic stem cells and HeLa cells. In our study, the cumulative curve indicated that the proportion of circRNAs not modified by m^6^A was greater than that of circRNAs modified by m^6^A, this may suggest that methylation downregulates circRNA expression. However, the potential effects of RNA modifications not only depend on the m^6^A quantity but also on the percentage of transcripts that are modified (Gilbert et al., 2016). Thus, we researched modifications and expression patterns of the circRNA and found 4 patterns including 96 circRNAs with both changed m^6^A quantity and m^6^A methylation level. The relationships between the expression and m^6^A modification of circRNAs in these four patterns were different. We also found 897 circRNAs with hypomethylated level but with no changes in m^6^A quantity. The changes in their m^6^A levels may be additional effects caused by their expression changes and have no matter with m^6^A modification. Therefore, we propose that when studying the role of m^6^A in circRNAs, both the m^6^A quantity and the methylation level of this circRNA need to be considered.

As circRNAs could sponge miRNA and affect its effect on targeted genes, many miRNAs (Wang et al., 2016, 2017; Huang et al., 2019a) and genes (Dumas and Pollack, 2008) have been researched in MT. CircRNAs with differences in both m^6^A quantity and m^6^A level were selected to construct circRNA–miRNA networks. We found several important miRNAs that participated in the pathological process could bound these m^6^A circRNAs, including miR-873a-5p, miR-103-1-5p, and miR-107-5p. These results suggest that m^6^A modification of circRNAs may influence the interactions between circRNAs and miRNAs. CircRNA 007895, circRNA 006829, and circRNA 004940 were predicted to bind miR-103-1-5p and miR-107-5p. However, little research has focused on these circRNAs. It will be necessary to investigate how m^6^A-modification regulates them in the pathological process of MT.

Finally, some limitations of our study should be acknowledged. First, we only detected the RNA expression of m^6^A-related enzymes and did not confirm the results based on the protein and enzymatic activity levels. In addition, the precise mechanism of ALKBH5 and YTHDF1 during MT needs to be studied. Second, we only focused on the global situation and did not explore the specific function of m^6^A-circRNAs in the development of MT. Further *in vivo* and *in vitro* research will be carried out to investigate the m^6^A-circRNA-mediated precise regulatory mechanisms in MT.

## Conclusion

This study is the first to reveal the m^6^A methylation pattern of circRNAs in the spinal cord of morphine-tolerant rats and provides a deeper understanding of the functional role of circRNAs. The results may bring novel clues for epigenetic etiology and pathogenesis of MT.

## Data availability statement

The datasets presented in this study can be found in online repositories. The names of the repository/repositories and accession number(s) can be found in the article/Supplementary material.

## Ethics statement

The animal study was reviewed and approved by the Central South University Ethics Committee (ethical no. 202103141).

## Author contributions

MX and WZ conceived and designed the experiments, performed the experiments, analyzed the data, and wrote the manuscript. YS, MD, JD, and TD performed animal experiments and analyzed the data. MX and ZS completed the bioinformatics analysis. WZ revised the manuscript. All authors read and approved the final manuscript.

## Footnotes

1
https://david.ncifcrf.gov/

2
https://www.omicstudio.cn/tool/43

## Supplementary material

Supplementary Figure 1 Distribution of circRNAs from the perspective of m^6^A-methylation level. **(A)** Hierarchical clustering shows global circRNAs m^6^A- methylation level between the MT group and NS group (*n* = 4). **(B)** The volcano plot shows the distribution of circRNAs with differentially methylation levels (Fold change ≥ 1.5 and *P* < 0.05) between the MT group and the NS group. **(C)** The nine-quadrant graph shows the relationship between the m^6^A methylation level and the expression of circRNAs.

## Abbreviations

m^6^A: N6-Methyladenosine
circRNA: circular RNA
ncRNAs: non-coding RNAs
miRNA: microRNAs
lncRNAs: long non-coding RNAs
METTL: Methyltransferase-like
WTAP: WT1 associated protein
YTHDC: YTH domain-containing
FTO: obesity-associated protein
ALKBH5: AlkB homologue 5
MT: Morphine tolerance
NS: Normal saline
%MPE: percent of the maximum possible effect
MeRIP: Methylated RNA immunoprecipitation
qRT-PCR: Quantitative real-time polymerase chain reaction
DE: different methylated
GO: Gene Ontology
KEGG: Kyoto Encyclopedia of Genes and Genomes
BP: Biological process
CC: Cellular component
MF: Molecular functions.
