# Supplementary figures and images for: Identification and characterization of N6-methyladenosine circular RNAs in the spinal cord of morphine-tolerant rats
Source: Front Neurosci. 2022 Aug 5;16:967768. doi: 10.3389/fnins.2022.967768 (PMC9388936; doi:10.3389/fnins.2022.967768)

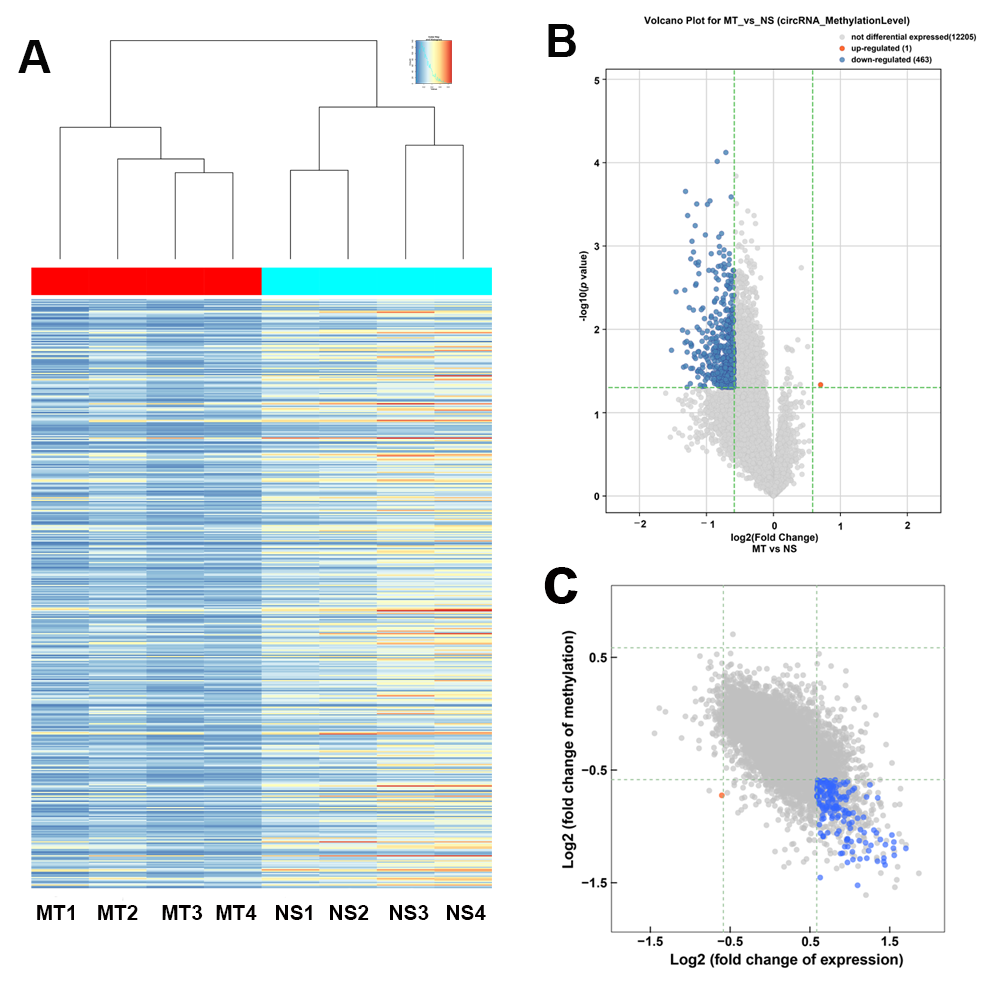

Supplement: Supplementary Figure 1 — Distribution of circRNAs from the perspective of m6A-methylation level. (A) Hierarchical clustering shows global circRNAs m6A- methylation level between the MT group and NS group (n = 4). (B) The volcano plot shows the distribution of circRNAs with differentially methylation levels (Fold change ≥ 1.5 and P < 0.05) between the MT group and the NS group. (C) The nine-quadrant graph shows the relationship between the m6A methylation level and the expression of circRNAs. [file Image_1.TIF]
