# Supplementary material for: Identification and characterization of N6-methyladenosine circular RNAs in the spinal cord of morphine-tolerant rats
Source: Front Neurosci. 2022 Aug 5;16:967768. doi: 10.3389/fnins.2022.967768 (PMC9388936; doi:10.3389/fnins.2022.967768)
Supplement: Supplementary file 2 [file Table_1.DOCX]

Supplementary Table 1: Primers used for real-time PCR.

| Name | Primer' sequence (5'-3') | Product size (bp) |
| --- | --- | --- |
| GAPDH | F: GCTCTCTGCTCCTCCCTGTTCTA | 124 |
|  | R: TGGTAACCAGGCGTCCGATA |  |
| METTL3 | F: TTGACTACAGTGGCTACCTTT | 220 |
|  | R: CCTTGGCTGTTGTGGTATT |  |
| METTL14 | F: GAGTATGTTTGCGAAAGTGGG | 84 |
|  | R: TTGTCTTTCCAGGATTGTTCTT |  |
| WTAP | F: GAAAAACTAAAGCAGCAACAG | 267 |
|  | R: CGTAAACTTCCAGGCACTC |  |
| FTO | F: GAGCGGGAAGCTAAGAAA | 100 |
|  | R: GCTGCCACTGCTGATAGAA |  |
| ALKBH5 | F: TTAGCGACTCGGCACTTT | 127 |
|  | R: TCATCAGCAGCATACCCAC |  |
| YTHDC2 | F: TGTAGGAGAAACTGGGTCTG | 255 |
|  | R: GTACGAAGCAATACCCCATTAG |  |
| YTHDF1 | F: ACAGTTACCCCTCGATGAGTG | 128 |
|  | R: GGTAGTGAGATACGGGATGGGA |  |
| YTHDF2 | F: GAGCAGAGACCAAAAGGTCAAG | 102 |
|  | R:CTGTGGGCTCAAGTAAGGTTC |  |
| rno_circRNA_012088 | F: TTGTTTGGTGGCTGGGATGG | 202 |
|  | R: AGCAGGCCTTTCAAGCTTCA |  |
| rno_circRNA_006829 | F: TTTGGATACCTGTGGCCAGT | 103 |
|  | R: AGCTCGCCTTTAGAGTGCAG |  |
| rno_circRNA_004940 | F: TCAGTTTGCCTCCCCAATGA | 101 |
|  | R: TCGCAAGGTCAAAGGACTGT |  |
| rno_circRNA_002718 | F: GTTCGGCATAGGGGACGG | 104 |
|  | R: GGCTCCAGTGTCATTGGCT |  |
